# Residual cancer is a strong predictor of survival in T3 incidental gallbladder cancer

**DOI:** 10.1186/s12893-022-01869-5

**Published:** 2022-12-28

**Authors:** Chuan You, Mengyi Xie, Meng Ling, Facai Yang, Yinghe Qiu, Jingdong Li

**Affiliations:** 1 Department of Hepatobiliary Surgery, Hepatobiliary and Pancreatic Minimally Invasive Technology Laboratory, Affiliated Hospital of North Sichuan Medical College, Hepatobiliary and Intestine Research Institute, North Sichuan Medical College, No. 1 Maoyuan South Road, Shunqing District, Nanchong, 637000 Sichuan China; 2 Department of Organ Transplant, Eastern Hepatobiliary Surgery Hospital, Naval Military Medical University, Shanghai, 200438 China

**Keywords:** Incidental gallbladder cancer, T3 stage, Residual cancer, Adjuvant chemoradiotherapy

## Abstract

**Background and purpose:**

Index cholecystectomy is insufficient for curing T3 incidental gallbladder cancer (IGC), and once residual cancer (RC) is found, the prognosis is often poor. The purpose of this study was to investigate the effect of RC on the prognosis and the optimal choice of adjuvant therapy for R0 reresection patients with T3 IGC.

**Methods:**

We retrospectively reviewed data from patients with T3 IGC who underwent radical reresection from January 2013 to December 2018. RC was defined as histologically proven cancer at reresection. Demographics and tumour treatment-related variables were analysed in correlation with RC and survival. Adjuvant (Adj) chemoradiotherapy (CRT) was correlated with overall survival (OS) and disease-free survival (DFS).

**Results:**

Of the 167 patients with IGC who underwent surgery, 102 underwent radical extended resection. Thirty-two (31.4%) RCs were found. Hepatic side tumours (T3h) and both side tumours (T3h + T3p) were associated with the presence of RC. In multivariate analysis, RC and lymph node metastasis were independent prognostic factors for DFS and OS (P < 0.05). RC was associated with a significantly shorter median OS (20 vs. 53 months; P < 0.01) and DFS (11 vs. 40 months; P < 0.001) despite R0 resection. For R0 reresection patients with RC and/or lymph node metastasis, Adj CRT significantly improved OS (P = 0.024).

**Conclusion:**

Residual cancer and lymphatic metastasis are important factors for the poor prognosis of T3 IGC despite R0 resection, and these patients should actively receive adjuvant therapy.

**Supplementary Information:**

The online version contains supplementary material available at 10.1186/s12893-022-01869-5.

## Introduction

Gallbladder cancer (GBC) has an insidious onset, high malignancy and poor overall prognosis. Only one-fifth of patients are diagnosed early, and 80% are diagnosed with advanced disease [1]. Approximately half of the patients are found incidentally during or after elective or emergency cholecystectomy [2]. Laparoscopic cholecystectomy has been widely accepted as the gold standard for the surgical treatment of benign gallbladder disease, but with the application of laparoscopic techniques, especially in community hospitals, the diagnosis of IGC has greatly increased [3]. Index cholecystectomy is insufficient for curative treatment of IGC staged above T1b. A study suggested that RC is associated with poor outcomes in IGC [4]. IGC is curable after R0 extended resection, but definitive resection (including cholecystectomy, partial hepatectomy, and lymphadenectomy) is attained in only 43–63% of patients, and approximately 10–76% of these patients were found to have RC with the most common sites being the gallbladder fossa and lymph nodes [5–7]. Patients with T3 stage disease have the highest incidence of GBC, perineural and/or lymphovascular invasion, and the T3 stage is strongly associated with RC [7]. Studies have shown that surgical resection combined with adjuvant therapy can significantly improve the 3-year OS of GBC patients, but only approximately 30% of patients receive adjuvant chemotherapy and/or radiotherapy after surgery [8]. To date, the need for adjuvant therapy after R0 extended resection remains controversial. Which patients can benefit from adjuvant therapy? This remains a question worth exploring. Therefore, the purpose of this study was to investigate the effect of RC on the prognosis and the optimal choice of adjuvant therapy for R0 reresection patients with T3 IGC (Additional file 1: Table S1).

## Materials and methods

### Study population

**Fig. 1:**
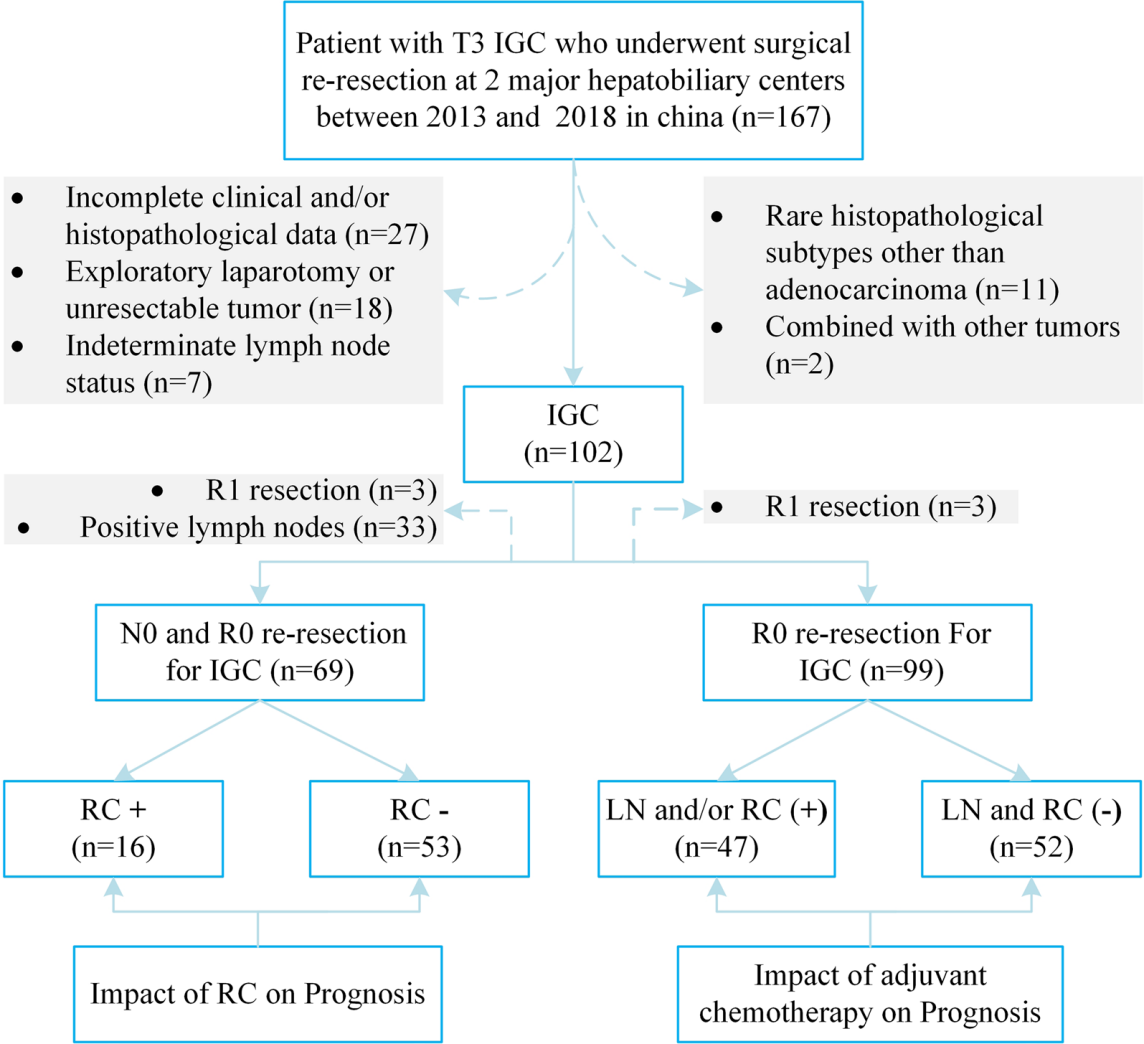
Consort diagram. IGC, incidental gallbladder cancer; RC−, absence of residual cancer; RC + , presence of residual cancer; LN/RC (−), absence of residual cancer and lymph node-negative; LN/RC ( +), presence of residual cancer and/or lymph node-positive

Clinicopathological data of 167 patients with T3 GBC who underwent radical extended resection were collected from 2 major hepatobiliary centres between January 2013 and December 2018; Eastern Hepatobiliary Surgery Hospital, Second Military Medical University (Shanghai, China, n = 87), Affiliated Hospital of North Sichuan Medical College (Nanchong, China, n = 80). A clinical and histopathological diagnosis of T3 IGC and age ≥ 18 years old were used as inclusion criteria. To assess prognostic factors for IGC, we excluded the patients with the following characteristics: those with incomplete clinical and/or histopathological data (n = 18), those with exploratory laparotomy or unresectable tumours (n = 18), those with an indeterminate lymph node status (n = 7), those that had IGC combined with other tumours (n = 2), and those with rare histopathological subtypes other than adenocarcinoma (n = 11). Finally, 102 patients were included in this study. See Fig. 1. The study complied with the requirements of the Declaration of Helsinki, and the patients and their families signed informed consent for the operation. The experimental protocol was approved by Ethics Committee of affiliated Hospital of North Sichuan Medical College.

### Definitions

IGC was defined as a gallbladder malignancy confirmed by histopathological examination after index cholecystectomy of a gallbladder disease initially diagnosed preoperatively or intraoperatively as benign. The “abdominal and hepatic sides” were defined according to the procedure of Shindoh J and colleagues [9]. RC is defined as tumour invasion confirmed by pathology at the liver, bile duct or adjacent tissue or organs after radical extended resection. R0 resection was defined as resection with macroscopically and microscopically tumour-free margins, and R1 resection was defined as resection with microscopically positive margins.

### Resection procedures

IGC patients from different centres all underwent radical extended resection within 1–2 months after index cholecystectomy. The specific re-excision procedure is as follows: (1) Preoperative imaging assessment (PET-CT examination if necessary) suggested that the tumour was resectable, without distant and intra-abdominal metastasis; (2) Laparoscopy or laparotomy was performed after successful anaesthesia to redetermine the resectability of the tumour; (3) Radical resection of IGC included liver wedge resection or S4b + S5 segment liver resection or extensive liver resection (≥ 3 liver segments), resection of involved tissues or organs around the gallbladder, and regional lymphadenectomy; (4) The scope of regional lymphadenectomy included lymph nodes around the cystic duct and the hepatoduodenal ligament.

### Follow-up

Clinical staging of IGC was performed by the American Joint Committee on Cancer (AJCC) staging system (8th edition). Postoperative adjuvant therapy included radiotherapy or chemotherapy, and the chemotherapy regimen included capecitabine alone and gemcitabine combined with oxaliplatin or tegafur. OS was defined as the time from the date of radical surgery to the date of death from any cause or the date of the last follow-up. DFS from the date of radical resection to the date of recurrence or last follow-up.

OS was obtained by querying the patient's registered date of death, which was the most effective evaluation result of this study. DFS was obtained by screening serum CA199 levels and imaging to assess any suspected recurrent disease every three months for 2 years after surgery and every 6 months for > 2 years. The patient follow-ups started on the day of surgery and ended when the patient died or in December of 2019.

### Statistical analysis

SPSS 22.0 statistical software was used for analysis. Continuous variable data were expressed as the mean ± standard deviation or mean (interquartile range, IQR), and the comparison between groups was performed using the independent samples t test or Wilcoxon rank-sum test. Enumeration data were compared using the χ^2^ test or Fisher's exact test. Survival analysis was performed using the Kaplan–Meier method, and survival rates were compared using the log-rank test. Multivariate analysis was performed using the Cox proportional hazards model. P < 0.05 was regarded as a statistically significant difference.

## Results

### Clinicopathological features

RC was found in 31.4% (32/102) of IGC patients after index cholecystectomy, with liver RC being the most common (81.3%, 26/32), followed by bile duct RC (18.8%, 6/32) and adjacent tissue or organ RC (9.4%, 3/32). The original tumour location was closely related to RC. The RC in patients with peritoneal-side tumours (T3p) was significantly lower than in patients with T3h or T3h + T3p (P < 0.05). Preoperative CA199 levels [36.3 (7.2–212.9) vs. 11.8 (6.7–20.7) U/ml, P = 0.027], surgery margin positivity (9.4% vs. 0%, P = 0.029), poorly differentiated tumour (40.6% vs. 17.1%, P < 0.05), lymph node positivity (46.9% vs. 25.7%, P = 0.034) and postoperative hospital stay [11 (9–15) vs. 10 (7–11) days, P = 0.034] in RC + patients were higher than those in RC− patients. The number of RC + patients undergoing extended cholecystectomy was significantly lower than that of RC− patients during laparoscopic cholecystectomy (15.6% vs. 40.0%, P = 0.022). There was no significant difference in other clinicopathological factors between the two groups. See Table 1.

**Table 1:** Comparison of Clinicopathologic Variables between Patients with RC− versus RC +

Characteristics	All (n = 102)	RC + (n = 32)	RC− (n = 70)	P value
Sex, n (%)				
Male	43 (42.2)	15 (46.9)	28 (40.0)	0.514
Female	59 (57.8)	17 (53.1)	42 (60.0)	
Age, mean ± SD, year	59 ± 11	61 ± 9	59 ± 11	0.271
CA19-9, median (IQR), U/ml	12.4 (6.8–40.1)	36.3 (7.2–212.9)	11.8 (6.7–20.7)	0.027
First cholecystectomy procedure				
Extended cholecystectomy	33 (32.4)	5 (15.6)	28 (40.0)	0.022
Simple cholecystectomy	69 (67.6)	27 (84.4)	42 (60.0)	
Tumour location*, n (%)				
Peritoneal side	76 (74.5)	6 (18.8)	70 (100)	< 0.001
Hepatic side	23 (22.5)	23 (71.9)	0	
Both sides	3 (2.9)	3 (9.4)	0	
Surgical approach, n (%)				
Radical resection	93 (91.2)	28 (87.5)	65 (92.9)	0.456
Extended resection	9 (8.8)	4 (12.5)	5 (7.1)	
Operation duration, mean ± SD, h	3.5 (3.0–4.0)	4.0 (3.5–4.5)	3.5 (3.0–4.0)	0.090
Intraoperative blood loss, median (IQR), ml	300 (200–400)	300 (200–450)	300 (188–400)	0.166
Surgical Margin				
Positive	3 (2.9)	3 (9.4)	0	0.029
Negative	99 (97.1)	29 (90.6)	70 (100)	
Peripheral neurovascular invasion, n (%)	11 (9.8)	6 (18.8)	5 (7.1)	0.079
No. of positive lymph node, median (IQR)	2 (1–4)	2 (1–4)	2 (1–3)	0.682
No. of harvested lymph node, median (IQR)	5 (4–8)	6 (3–9)	5 (4–7)	0.166
Tumour differentiation grade, n (%)				
Well	8 (7.9)	4 (12.1)	4 (5.7)	0.010
Moderately	69 (67.7)	15 (46.9)	54 (77.1)	
Poorly	25 (24.5)	13 (40.6)	12 (17.1)	
Lymph node status				
Negative	69 (67.6)	17 (53.1)	52 (74.3)	0.034
Positive	33 (32.4)	15 (46.9)	18 (25.7)	
TNM Group of AJCC 8th*, n (%)				
IIIA	69 (67.6)	17 (53.1)	52 (74.3)	0.079
IIIB	24 (23.5)	10 (31.3)	14 (20.0)	
IVB	9 (8.8)	5 (15.6)	4 (5.7)	
Adjuvant chemoradiotherapy				
Have	22 (21.6)	6 (18.8)	16 (22.9)	0.640
None	80 (78.4)	26 (81.3)	54 (77.1)	
Postoperative major complications*, n (%)	8 (7.8)	5 (15.6)	3 (4.3)	0.104
Postoperative hospital stay, mean ± SD, d	10 (8–12)	11 (9–15)	10 (7–11)	0.034

*Complications of Clavien–Dindo classification grade III or above; *IQR* interquartile range

### Residual cancer associated with poor prognosis in IGC

To analyse the risk factors for the prognosis of IGC, we performed univariate and multivariate analyses for 102 IGC patients. The results showed that RC (HR = 3.057, 95% CI: 1.276–7.326, P = 0.012), intraoperative blood transfusion (HR = 0.368, 95% CI: 0.149–0.909, P = 0.030) and lymph node metastasis (HR = 2.263, 95% CI: 1.137–4.504, P = 0.020) were independent risk factors affecting OS; RC (HR = 3.165, 95% CI: 1.572–8.314, P = 0.002), intraoperative blood transfusion (HR = 2.396, 95% CI: 1.046–5.492, P = 0.030) and lymph node metastasis (HR = 3.316, 95% CI: 1.784–5.601, P < 0.001) were independent risk factors for DFS. See Table 2.

**Table 2:** Univariable and multivariable analysis for OS and DFS among all IGC patients (n = 102) with definitive surgical treatment

Risk factor	OS				DFS			
	Univariate		Multivariate		Univariate		Multivariate	
	HR (95% CI)	P value	HR (95% CI)	P value	HR (95% CI)	P value	HR (95% CI)	P value
Age (≥ 60 years)	1.477 (0.844–2.585)	0.172			1.425 (0.859–2.346)	0.171		
CA19-9 (≥ 39 U/mL)	2.615 (1.433–4.739)	0.002	2.114 (1.041–4.292)	0.038	2.311 (1.323–4.038)	0.003	1.402 (0.768–2.559)	0.271
Residual cancer (Absent vs. Present)	4.650 (2.647–8.171)	< 0.001	3.347 (1.439–7.785)	0.005	3.440 (2.048–5.779)	< 0.001	3.615 (1.572–8.314)	0.002
Tumour location (vs. Peritoneal side)		< 0.001		0.119		0.001		0.132
Hepatic side	2.388 (1.302–4.382)	0.005	0.566 (0.211–1.523)	0.260	2.152 (1.227–3.772)	0.007	0.495 (0.199–1.231)	0.130
Both sides	9.429 (3.113–28.559)	< 0.001	2.559 (0.638–10.260)	0.185	6.204 (1.849–20.816)	0.003	1.431 (0.353–5.800)	0.616
Intraoperative blood transfusion (Absent vs. Present)	2.346 (1.175–4.685)	0.016	3.068 (1.274–7.389)	0.012	1.915 (0.906–4.049)	0.089	2.396 (1.046–5.492)	0.039
R1 resection and/or peri neural and/or lymphovascular invasion (Absent vs. Present)	2.792 (1.343–5.805)	0.006	0.905 (0.322–2.542)	0.849	1.693 (0.674–4.256)	0.263		
Lymph node (Negative vs. Positive)	3.440 (1.957–6.047)	< 0.001	2.783 (1.503–5.156)	0.001	3.175 (1.898–5.310)	< 0.001	3.316 (1.784–5.601)	< 0.001
Differentiation grade (High/Intermediate vs. Low)	2.280 (1.261–4.123)	0.006	1.946 (0.948–3.993)	0.070	1.467 (0.827–2.602)	0.190		

**Fig. 2:**
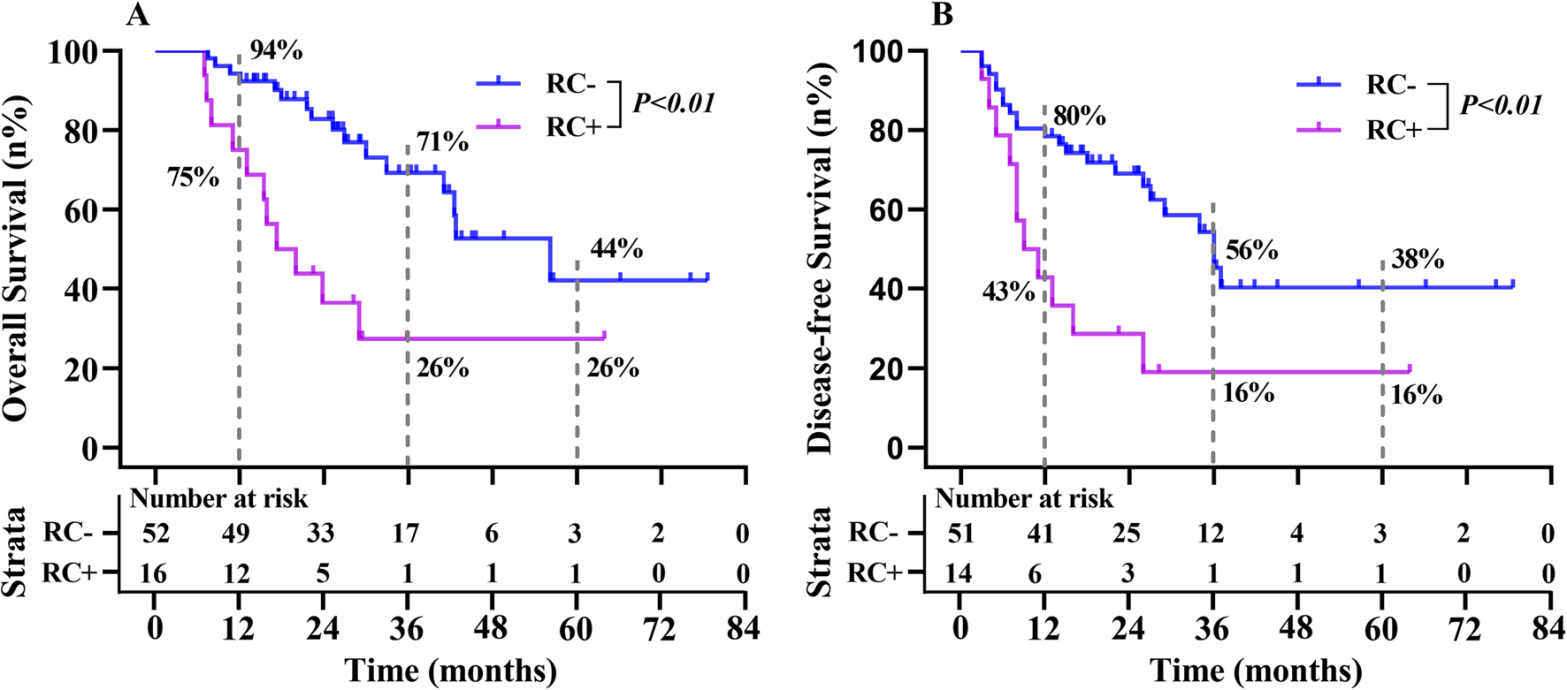
Five-year OS and DFS for IGC patients with N0 and R0 according to the presence of RC. *N0* lymph node-negative, *R0* surgical margin negative, *RC* residual cancer. RC (−), no residual cancer; RC (+), have residual cancer. The 1-, 3-, 5-years OS and DFS for RC(-) and RC(+) patients are 94% vs 75%, 71% vs 26%, 44% vs 26%，and 80% vs 43%, 56% vs 16%, 38% vs16%，respectively

We excluded patients with lymph node metastasis (n = 33) and R1 resection (n = 3) to analyse the impact of RC on the prognosis of patients with IGC. The median follow-up was 25 (7–79) months [19 (7–64) months in patients with RC + versus 26 (8–79) months in patients with RC−]. RC + was associated with a significant reduction in OS [20 months (RC +) vs. 53 months (RC−), p < 0.01] and DFS [11 months (RC +) vs. 40 months (RC−), p < 0.01], as shown in Fig. 2.

### Adjuvant chemoradiotherapy

**Fig. 3:**
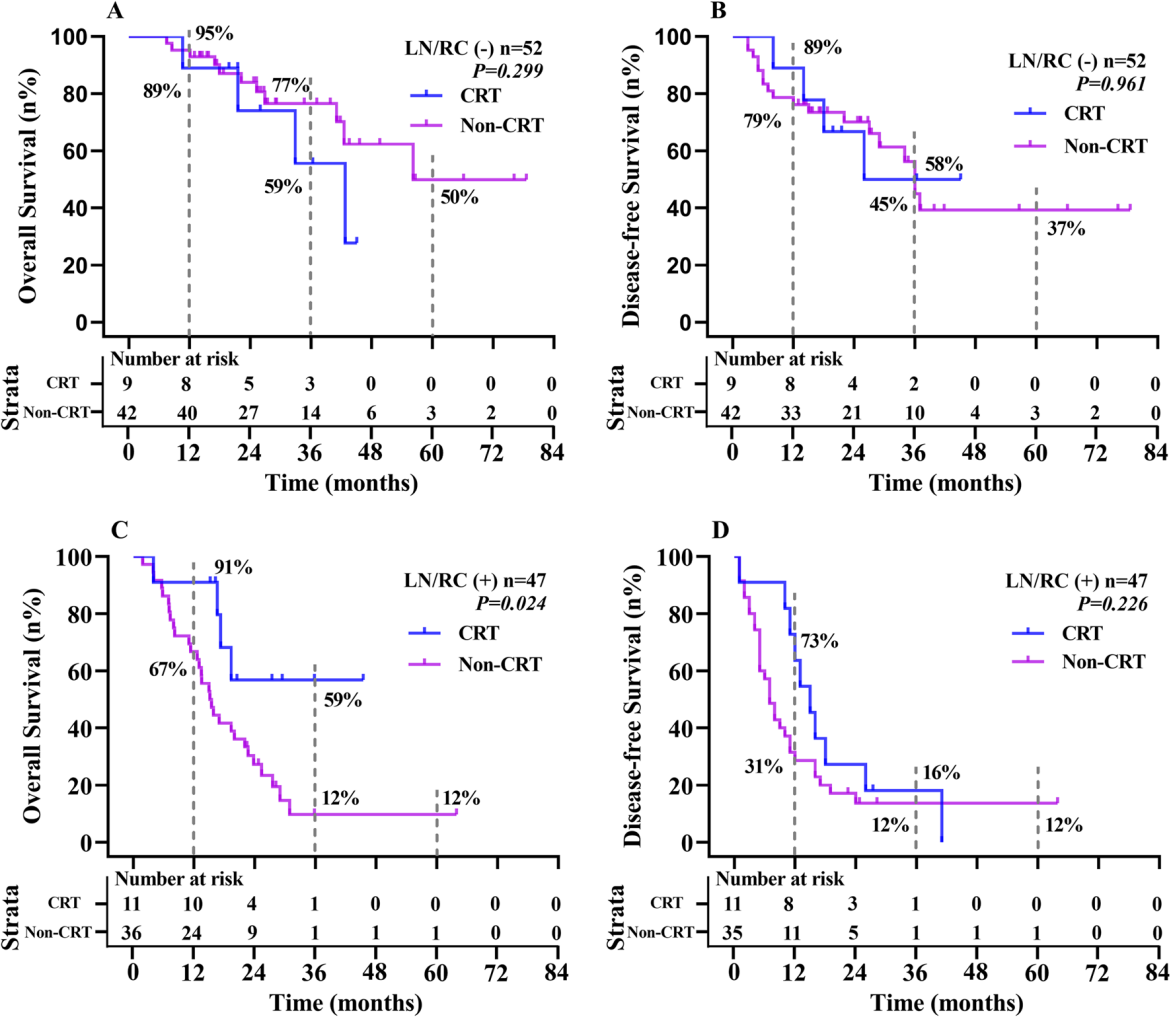
Five-year OS and DFS for IGC patients with LN/RC (−) and LN/RC ( +) according to the presence of adjuvant chemoradiotherapy. *LN/RC (−)* lymph node-negative and no residual cancer, *LN/RC ( +)* lymph node-positive and/or residual cancer; *CRT* chemoradiotherapy. The 1-, 3-, 5-years OS and DFS for CRT and non-CRT with LN/RC (−) patients are 89% vs 95%, 59% vs 77%, NC vs 50%，and 89% vs 79%, 58% vs 45%, NC vs 37%，respectively; The 1-, 3-, 5-years OS and DFS for CRT and non-CRT with LN/RC (+) patients are 91% vs 67%, 59% vs 12%, NC vs 12%，and 73% vs 31%, 16% vs 12%, NC vs 12%，respectively

To analyse the benefit for R0 reresection patients of IGC with adjuvant CRT, we excluded 3 patients with R1 resection. Among the LN/RC (-) (lymph node-negative and no residual cancer) and LN/RC ( +) (lymph node-positive and/or residual cancer) patients, 10 (10/52, 19.2%) and 11 (11/47, 23.4%) received adjuvant radiotherapy or chemotherapy, respectively. Kaplan–Meier analysis showed that LN/RC (-) patients did not benefit from adjuvant CRT in terms of OS or DFS (all P values > 0.05). Compared with surgical resection alone, adjuvant CRT combined with surgical resection significantly improved the median OS of patients with LN/RC ( +) (Adj CRT vs. Non-Adj CRT = 36 vs. 17 months, P = 0.024), while the median DFS (Adj CRT vs. Non-Adj CRT = 18 vs. 9 months, P = 0.226) was not significantly different. See Fig. 3.

## Discussion

Gallbladder cancer is a rare malignant tumour of the digestive system, but its incidence has increased in recent years, with a higher incidence in women than in men [10]. For early-stage gallbladder cancer, achieving R0 resection is the key to planning for surgical treatment, while for advanced disease, comprehensive multidisciplinary management is emphasised. Index cholecystectomy may lead to RC, and no assessment of lymph node status, reassessment of tumour staging and removal of the residual disease have a positive effect on improving patient outcomes [2, 11]. Although extended resection is recommended for selected IGC patients without the disseminated disease, optimal management of IGC remains a critical issue [4, 6, 7, 12, 13]. The main reason is that residual disease detection represents a poor outcome regardless of anatomical location.

An international multicentre study in 2015 showed that patients with T2h had higher vascular invasion, neural invasion, and lymph node metastasis than patients with T2p, and T2h were often associated with worse prognosis [9]. The gallbladder adjacent to the hepatic side has no serosal layer, there are as many as 20 (2–20) gallbladder veins, and the tumour easily metastasises to the liver bed through the gallbladder veins [14]. RC was found in 31.4% of patients after index cholecystectomy, with the liver being the most common residual site. In addition, patients with RC had higher rates of lymph node positivity and lower differentiation tumours. These are the possible reasons for the poor prognosis of RC patients.

Although surgical resection has an irreplaceable role in managing IGC, surgical treatment alone is unlikely to be sufficient in patients with RC or stage III-IV disease, and systemic treatment has an important emerging role [15, 16]. However, systematic reviews and meta-analyses pointed out that there is no strong evidence that adjuvant therapy is effective in GBC patients undergoing radical resection and only has some benefit in patients with positive lymph node disease, and positive surgical margins, or advanced disease [17, 18]. A recent cohort study of 6391 GBC patients who underwent definitive surgery from the US National Database (NCBD) showed that 49.2% received adjuvant chemotherapy and 1.6% received neoadjuvant chemotherapy [19]. Further propensity score matching analysis suggested that GBC patients who received adjuvant chemotherapy had a longer median OS than those who received surgery alone (22 vs. 18 months, HR = 0.78, 95% CI: 0.63–0.96), and lymph node-positive patients benefited from neoadjuvant therapy (median OS: 30 vs. 14 months, P = 0.002).

Our analysis of prognostic factors in 102 IGC patients who underwent re-excision showed that lymph node-positive status and RC were independent risk factors for poor OS and DFS. Even with R0 resection, patients with lymph node positivity and/or RC were associated with low median OS and DFS. Recurrent disease is the main reason for the poor prognosis; 84% of GBC patients experience recurrence within the first 18 months after radical resection, of which 53% only have a local recurrence, 26% only have a distant recurrence, 21% have a local and distant recurrence, and the liver is the most common site, followed by carcinomatosis and lymph nodes [20]. In a subgroup analysis, we found that adjuvant chemotherapy significantly improved OS for IGC patients with lymph node metastases and/or residual disease after R0 resection. Therefore, we recommend routine adjuvant therapy for T3 IGC patients with residual disease or lymphatic metastasis.

Certainly, the research has some deficiencies that need to be rectified and optimized in the researches in the future. Firstly, the diagnosis of T3 IGC may be influenced by artificial subjective factors, the main reason is that the accuracy of the diagnosis depends heavily on the experience of the clinician and pathologist. Second, the number of patients who received adjuvant chemoradiotherapy after R0 resection was limited, which was not enough to fully reflect the clinical value of adjuvant therapy. Finally, the sample size of this study was limited. Limited by retrospective studies, multi-center, large-sample and prospective studies can provide more evidence-based evidence in the future.

In conclusion, index cholecystectomy does not attain the goal of curative treatment of T3 IGC, and all patients undergoing index cholecystectomy should be systematically evaluated to determine the need for reoperation. If the underlying conditions permit, extended resection should be actively performed for all resectable IGC, and R0 resection should be as extensive as possible to attain better OS and DFS. Residual disease and lymphatic metastasis after index cholecystectomy are the main reasons for the poor prognosis of IGC, and adjuvant therapy should be actively used in these patients.

## Supplementary Information

**Additional file 1: Table S1.** T3 IGC clinicopathologic variables for data collection.

## Data Availability

The datasets generated and analysed during the current study are not publicly available due but are available from the corresponding author on reasonable request.
